# A functional PCR-CTPP marker targeting an intronic SNP in *OsLAC11* gene for detecting kernel smut resistance in rice

**DOI:** 10.3389/fpls.2026.1809531

**Published:** 2026-07-07

**Authors:** Zakaria Hossain Prodhan, Stanley Omar Pe Benito Samonte, Kimberly Suazo Ponce, Darlene Lonjas Sanchez, Xin-Gen Zhou, Sabin Khanal, Roland Bocco, Lloyd Theodore Wilson

**Affiliations:** 1 Texas A&M AgriLife Research and Extension Center, Beaumont, TX, United States; 2 Texas A&M University, College Station, TX, United States

**Keywords:** functional marker, PCR-CTPP marker, resistance genes, rice, rice kernel smut, *Tilletia horrida*

## Abstract

Rice kernel smut, caused by the fungus *Tilletia horrida* Tak., is an emerging disease of rice. The pathogen infects germ cells at the early flowering stage, yet the plant remains asymptomatic until maturity. The rice plant may recognize it as a stress-inducible factor, triggering the expression of various stress-responsive genes and pathways. However, researchers are attempting to identify kernel smut-resistant genes in cell-surface proteins (elicitors), secretory proteins, glycoside hydrolase family proteins, and effector proteins. Based on annotated effector proteins and integrated analyses of available gene-expression datasets, this study aims to identify allelic variation in a putative candidate gene to develop a functional marker for marker-assisted identification of both resistant and susceptible rice lines. The gene *OsLAC11* was found to be strongly associated with kernel smut resistance and is expressed on the first day of flowering in rice, which aligns with the infection window and supports its role in resistance. Sanger sequencing of the *OsLAC11* gene across ten rice germplasms with varying levels of kernel smut resistance identified two single nucleotide polymorphisms (SNPs) in intronic regions. The first SNP (G/T allele) was used to develop a functional marker using Polymerase Chain Reaction (PCR) with the Confronting Two-Pair Primers (CTPP). This marker effectively differentiated kernel smut-resistant rice lines from susceptible ones and was validated through phenotypic disease ratings. The molecular marker developed in this study represents a significant advancement in breeding for kernel smut resistance, providing a robust tool to accelerate genetic gains and streamline marker−assisted selection in rice improvement programs.

## 1 Introduction

Rice (*Oryza sativa* L.) is one of the most important crops, serving as a staple food for half of the world’s population (Khan et al., 2019; He et al., 2025; Rahmawati et al., 2026) and contributing more than 20% of total calories to global food consumption (Liu et al., 2026; Tong et al., 2026). Nonetheless, rice cultivation encounters increasing threats from both biotic and abiotic stresses, including fungi, bacteria, viruses, and the impacts of climate change, such as variability in rainfall and temperature, that compromise yield stability (Anami et al., 2020; Kumar et al., 2025; Biswas et al., 2026). Notably, fungi are responsible for substantial annual losses by inducing various diseases in numerous crops, for instance, kernel smut caused by the fungus *Tilletia horrida* Tak (Khanal et al., 2023). Rice kernel smut is a severe grain disease and poses a significant threat to rice production worldwide, including both long-grain and hybrid cultivars (Chen et al., 2016; Greer and Espino, 2024). Over the past decade, the incidence and severity of kernel smut have risen in the United States, posing a serious threat to rice production (Espino, 2019; Khanal et al., 2023). In 2021, a widespread outbreak of kernel smut was reported across rice-growing regions in Texas and southwestern Louisiana (Khanal et al., 2023). *T. horrida* infects rice flowers and colonizes the inner floral organs with mycelia, ultimately producing masses of dark powdery teliospores (Wang et al., 2018a, 2022). The teliospores serve as the overwintering structure and can survive for more than a year in soil and for at least three years on the host seed (Wang et al., 2022; Seethapathy, 2025). Infection by *T. horrida* remains asymptomatic until grain maturity, when black powdery spores become visible, leading to a substantial reduction in grain quality and yield (Wang et al., 2018a, 2022). *T. horrida* hyphae do not invade the cells and embryo sacs; consequently, stigma cells and ovaries undergo no visible changes and retain the normal ability of plasmolysis at 12 h post-inoculation. These findings suggest the infection strategy of *T. horrida* is unique and distinct from that of other flower-infecting fungi (Wang et al., 2022).

The genome sequence of *T. horrida*, comprising 23.2 Mb, encodes 7,729 predicted genes, of which 6,973 were supported by RNA-seq data (Wang et al., 2018a). Among these, 597 genes encoding secreted proteins were annotated, and several key effector genes linked to pathogenicity were identified. Within these secreted proteins, 131 were predicted to be candidate effectors (Wang et al., 2018a, 2018b). Genes and functions related to pathogenicity were examined, revealing that *T. horrida* has fewer carbohydrate-active enzymes and secondary metabolites, likely due to its unique infection and biotrophic lifestyle. Additionally, genes coding for secreted proteins, secondary metabolism enzymes, and those listed in the pathogen-host interaction gene database showed high expression during early infection, supporting their possible roles in pathogenicity (Kainat et al., 2025). In *T. horrida*, only two secretory proteins (smut_5844 and smut_2965) have demonstrated the capability to induce non-host cell death, and an immune response has been recognized as an effector. One of the identified potential host interactors, smut_5844, was previously mentioned as the laccase-10 protein (Wang et al., 2019).

Laccases, which are predominantly present in plants and fungi, include fungal laccases that participate in lignin degradation, stress response, and morphogenesis (Janusz et al., 2020; Westrick et al., 2024). Meanwhile, rice possesses approximately 30 laccase genes (*OsLAC*s), which are crucial for plant development and defense mechanisms. The *OsLAC*s are induced by various stresses, including salt, drought, hormones, and heavy metals (Liu et al., 2017; Ding et al., 2025). These *OsLAC* genes are primarily expressed in secretory pathways (cell wall and intercellular gaps) during early developmental stages of the endosperm, growing roots, and stems. Most *OsLAC* genes contain glycosylation sites required for protein glycosylation, which are essential for protein folding, stability, and cell wall construction (Liu et al., 2017). The expression level of *OsLAC10* increased following copper (Cu) treatment and is likely involved in lignin production. It also inhibits Cu uptake by roots, which is essential for *Arabidopsis* to develop tolerance to Cu (Liu et al., 2017; Perkins et al., 2020). The *LAC11* gene is involved in monolignol polymerization (Zhao et al., 2013; Perkins et al., 2020). Disrupting *LAC4*, *LAC11*, and *LAC17* concurrently almost stops lignin deposition and slows plant development (Zhao et al., 2013; Ding et al., 2025).

A gene that is highly expressed during *T. horrida* infection (Wang et al., 2019, 2020b) and is associated with broad-spectrum biotic stress responses, lignin deposition, and salicylic acid synthesis can be a promising candidate for developing functional markers. Functional markers from gene sequences are valuable for marker-assisted breeding, as they reliably distinguish alleles at a single locus and are fully diagnostic of the target trait, offering advantages over microsatellites or SSRs and random DNA markers (Zhou et al., 2018; Kumar et al., 2024). The functional markers based on single nucleotide polymorphisms (SNPs) are the most widely used DNA markers for identifying genomic regions linked to important traits, thus speeding up breeding (Iqbal et al., 2025b). As the most common type of variation in plant genomes, SNPs enable high-resolution genotyping with exceptional accuracy. Additionally, SNPs are more efficient and cost-effective than other marker types (Hasan et al., 2021; Kumar et al., 2024). There are also various functional markers from SNP typing techniques, such as sequencing, mass spectrometry, high-resolution melting, SNP chips, and the SNaPshot system, that are used to study genetic variation and to support improvements in breeding and disease resistance (Kitazawa et al., 2019; Yang et al., 2024). However, these methods are complex, labor-intensive, and costly for extensive breeding screening (Zhou et al., 2018; Shang et al., 2021). In contrast, derived cleaved amplified polymorphic sequence (dCAPS) is a simple technique for SNP identification. Nevertheless, it requires laborious post-amplification enzymatic cleavage (Kaundun et al., 2019). The kcompetitive allele-specific PCR (KASP) marker is widely used for SNP genotyping and uses fluorescence resonance energy transfer (FRET) and dyes to detect genetic differences at a specific locus (Dipta et al., 2024). At this point, the Polymerase Chain Reaction (PCR) with Confronting Two-pair Primers (CTPP) is a suitable alternative approach that is simple and requires four primers in a single PCR reaction. This technique identifies SNP types by varying product lengths, thereby making it an optimal choice for high-throughput SNP typing (Hamajima, 2001; Jamshidi and Yari, 2022).

The objective of this study was to develop a functional marker for the kernel smut-responsive gene (*OsLAC11*), aiming to distinguish rice lines genotypically for their resistance or susceptibility to kernel smut. The functional marker containing the SNP in the first intron (c.111 + 74G>T, representing the G/T allele) of the *OsLAC11* gene produced distinct bands and effectively distinguished resistant and susceptible rice cultivars using PCR-CTPP. The genotyping results were validated by pathogen inoculation and standard phenotypic disease-scoring methods. This functional marker can be directly applied in rice breeding programs to identify lines that are resistant to kernel smut.

## 2 Materials and methods

### 2.1 Plant material

Sixteen rice cultivars (**Supplementary Table 1**) were used in this study, and the seeds were obtained from the USDA-ARS Germplasm Resources Information Network (GRIN). Nine cultivars were selected for their proven resistance to kernel smut (Akhtar and Sarwar, 1988; Singh et al., 1998) and were used for Sanger sequencing to detect single-nucleotide polymorphisms (SNPs). These were also assessed for kernel smut resistance under field conditions at the Texas A&M AgriLife Research Center in Beaumont, TX, USA. ‘Nipponbare’ was utilized as a reference rice variety to validate the genotyping accuracy for sequencing and SNP detection (Chen et al., 2014). Cultivars 11 to 16 were selected from a 2-year (2023 and 2024) kernel smut resistance evaluation study at the Texas A&M AgriLife Research Center in Beaumont, TX, USA, and their disease ratings were used to validate the proposed markers for kernel smut resistance.

### 2.2 Plant management

For the genotypic study, rice plants were grown in black plastic pots (10.2 cm long, 10.2 cm wide, and 10.9 cm high) filled with a soil mixture (50% field soil and 50% garden soil), with three seedlings per pot. The pots were placed inside large white plastic tubs (210.8 cm long, 91.4 cm wide, and 35.6 cm high) in the greenhouse (30°04'03.7"N latitude, 94°17'32.1"W longitude, 5 m above sea level) at the Texas A&M AgriLife Research Center at Beaumont, Texas, USA. For the artificial inoculation in the field, rice seeds were sown in May 2024 and April 2025, following standard cultural management practices described in the 2023 Texas Rice Production Guidelines (Dou and Yang, 2023).

### 2.3 DNA extraction

Leaf samples (about 5 cm from the tips of the flag leaf) were collected from two-week-old seedlings of cultivars 1 to 10 (**Supplementary Table 1**) and sent to CD Genomics (Shirley, NY, USA) for Sanger sequencing, where the DNA was extracted using the Tiangen Plant DNA Isolation Kit (Tiangen Biotech, Beijing, China). Leaf samples (about 4 cm from the tips of the flag leaf) were also collected from the two-week-old seedlings of each of the 16 cultivars (**Supplementary Table 1**) and used for DNA extraction using the DNeasy Plant Pro Kit (Qiagen, Hilden, Germany) for PCR amplification in the genetics lab of the Texas A&M AgriLife Research Center in Beaumont, TX, USA, following standard protocol (Iqbal et al., 2025a, 2025b). DNA quantity and quality were assessed using a NanoDrop 8000 (Thermo Fisher Scientific, DE, USA).

### 2.4 Sanger sequencing method

The forward and reverse primers (**Supplementary Table 2**) for Sanger sequencing and PCR amplification were designed using the PrimerQuest Tool (Integrated DNA Technologies, Inc., CA, USA) to flank the target DNA region of the *OsLAC11* (*Os03g0273200*) gene, with the Nipponbare genome as the reference. The PCR amplification for Sanger sequencing was performed in a final mix of 25 µl containing template DNA, Truepol 2X PCR Mix for Microbiome (ABclonal RK20720), nuclease-free water, and forward and reverse primers. The PCR profile was set to 45 seconds of initial denaturation at 98 °C, followed by 27 cycles of 10 seconds at 98 °C, 60 seconds of annealing at 62 °C, 30 seconds of initial extension at 72 °C, and a 5-minute final extension at 72 °C. The PCR products were separated on a 2% agarose gel, stained with GelRed^®^ Nucleic Acid Gel Stain (Biotium, Inc., Fremont, CA, USA), and visualized using the Corning Gel Documentation System (Axygen, USA). The PCR products were purified by the GeneJET PCR Purification Kit (Thermo Fisher Scientific, Foster City, USA) to remove primers, dNTPs, and enzymes. The sequencing was performed by CD Genomics (Shirley, NY, USA). The sequencing reaction was carried out using BigDye Terminator v3.1 mix (Thermo Fisher Scientific, Foster City, USA), primers, sequencing buffer, and template DNA. The purified product was loaded onto an ABI 3730xl sequencer (Thermo Fisher Scientific, Foster City, USA) to separate DNA fragments by size and detect fluorescently labeled ddNTPs. The sequence electropherograms were analyzed for base calling and quality assessment using Chromas Lite ver. 2.6.5 (https://technelysium.com.au/wp/chromas/).

### 2.5 Sequence alignment and SNP selection

The flanking sequence obtained from different targeted regions of the *OsLAC11* gene was arranged according to the rice lines and aligned using MEGA Software (Kumar et al., 2016). Sequence alignment revealed two SNPs, and the first intronic SNP with a T/G variant was selected for marker development.

### 2.6 Marker design

**Figure 1:**
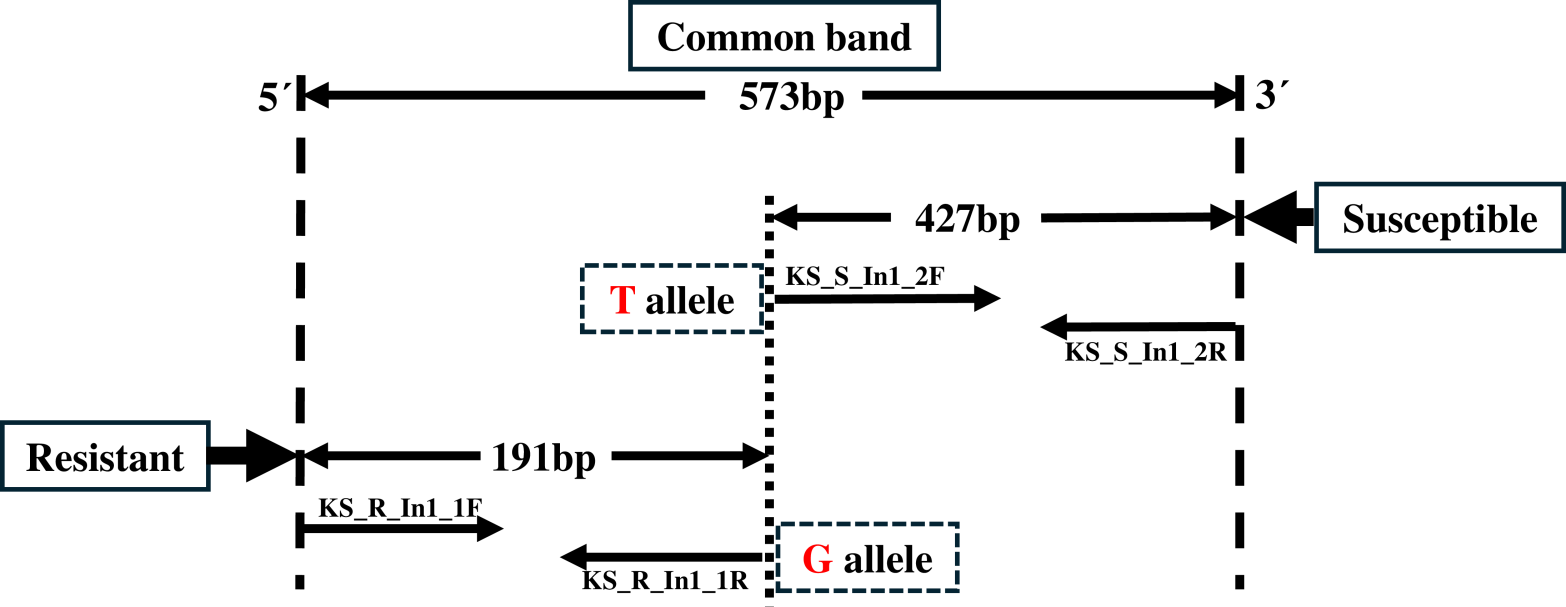
Design of the kernel smut marker. The 573 bp band is common and indicates successful amplification. The 427 bp band is specific to susceptible genotypes that have the T allele. The 191 bp band is specific to resistant genotypes that contain the G allele.

The functional marker for detecting kernel smut resistance in rice was developed using PCR-CTPP, targeting an SNP with T or G alleles (**Figure 1**). The primer (KS_S_In1_1R) for the G allele (antisense primer 1R) was designed to include C (antisense nucleotide for G) at the 3′ end, with the corresponding sense primer (1F) located upstream, resulting in a 191 bp amplified DNA fragment. Similarly, the sense primer that includes T (KS_S_In1_2F) and the antisense primer (KS_S_In1_2R) downstream of primer 2F were set to amplify the T allele, producing a 427 bp DNA fragment. The sizes of these two PCR products were distinct and could be differentiated by gel electrophoresis. Additionally, a 573-bp DNA fragment amplified between primers KS_S_In1_1F and KS_S_In1_2R confirmed successful amplification. The resistant genotype showed 191-bp and 573-bp bands, while the susceptible genotype displayed 427-bp and 573-bp bands.

### 2.7 Marker validation

Validation of markers for detecting kernel smut resistance via PCR was conducted in a 25 µl reaction mixture containing 12.5 µl of Quick-Load Taq 2X Master Mix (New England Biolabs, MA, USA), 100 ng of DNA, 0.50 µl of each of the four primers, and the remaining volume of RNase-Free Water. The leaf samples were collected from two-week-old seedlings of 11 to 16 cultivars (**Supplementary Table 1**) and amplified using a T100 Thermal Cycler System (Bio-Rad, USA) with a profile set to 5 min of initial denaturation at 94 °C, followed by 30 cycles of 30 sec at 94 °C, 30 sec of annealing at 61 °C, 30 sec of initial extension at 72 °C, and a 5 min final extension at 72 °C. The amplified PCR products were separated on 2% agarose gels (Amresco, USA), stained with GelRed^®^ Nucleic Acid Gel Stain (Biotium, Inc., Fremont, CA, USA), and visualized using the Corning Gel Documentation System (Axygen, USA). The marker validation experiments were repeated several times.

### 2.8 Inoculation and phenotypic evaluation of kernel smut

The *T. horrida* strains were collected in 2018 and 2019 from the affected rice field in the six major rice-growing states in the USA (Khanal et al., 2022) and were isolated from teliospores on 2% water agar, following the procedure described by (Chahal et al., 1993). Germination of teliospores was visually confirmed under a microscope after three days of incubation. Primary sporidia germinating from the single teliospores were transferred to potato dextrose agar (PDA) plates and incubated at 28 °C for 14 days to promote growth. Mycelium was placed in a solution comprising 2% sucrose and 20% glycerol at -80 °C for long-term storage (Khanal et al., 2022).

Monoteliosporidial cultures of the *T. horrida* strains were grown on PDA at 28 °C in an incubator for 14 days. Multiple strains belonging to each genetic group (Khanal et al., 2022) were mixed to form a fungal suspension, and a suspension of 10^5^ spores/ml was prepared in sterilized distilled water (Wang et al., 2020a). For each cultivar, 10 primary tillers were selected in each of three randomized plots and labeled with color tags for easy identification. The selected tillers were inoculated at the boot stage (5 to 7 days before heading) using a syringe, with 2 ml of spore suspension. Plants inoculated with sterilized distilled water served as controls. At the maturity stage of each cultivar, treated panicles were harvested, and the percentage of kernel showing kernel smut symptoms per panicle was assessed. Evaluations of field disease in the nursery were conducted at the Texas A&M AgriLife Research Center in Beaumont, Texas, USA, in 2023 and repeated in 2024 during the rice cropping season.

## 3 Results

### 3.1 Selection, structure, and sequence analysis of the kernel smut-resistant related gene

**Figure 2:**
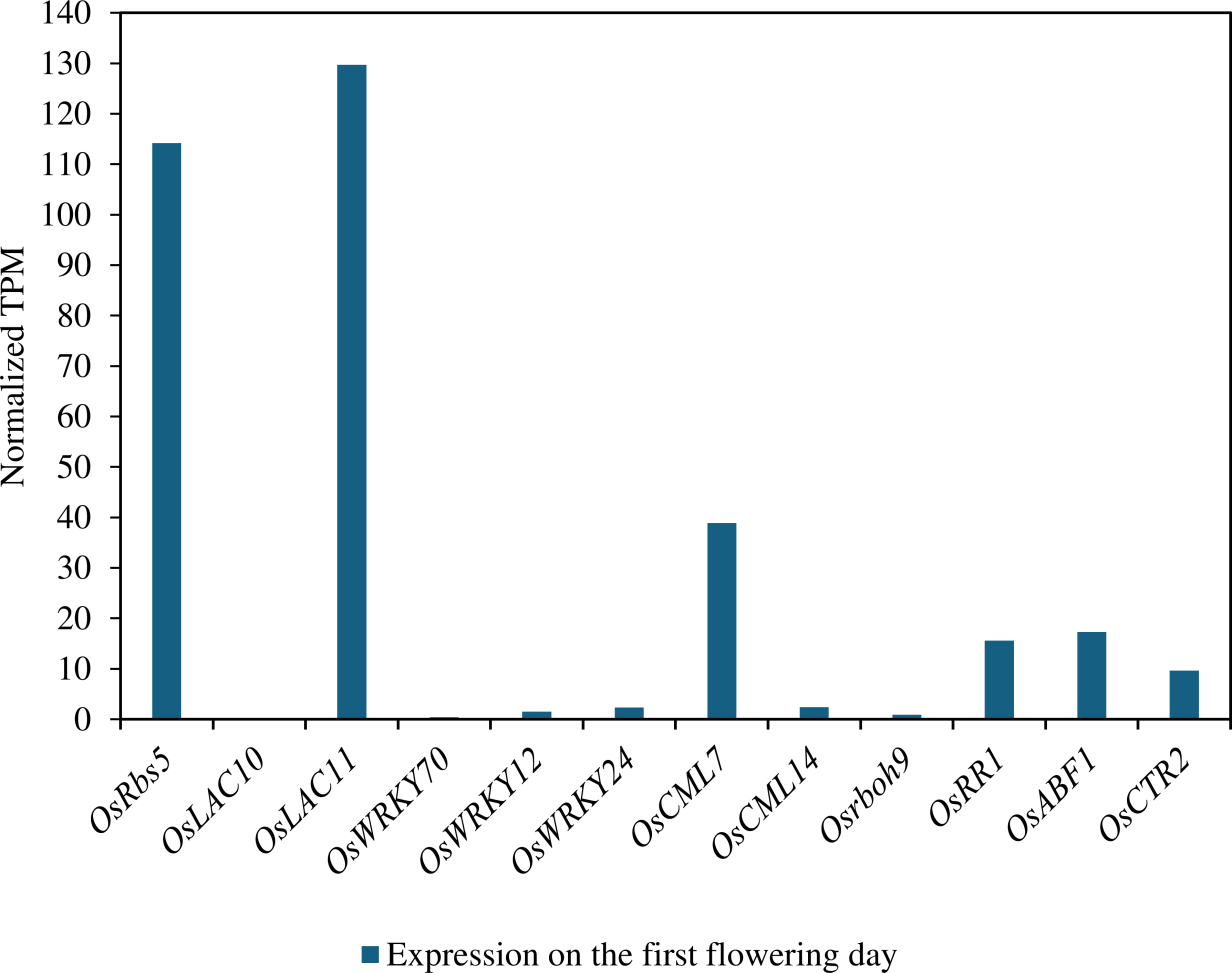
Expression analysis of kernel smut-related genes on the first flowering day. The normalized TPM (Transcripts Per Million) mean values were obtained from https://rapdb.dna.naro.go.jp/.

In the present study, all the previously reported genes related to kernel smut resistance (Wang et al., 2015, 2020b) were evaluated, and their genomic characteristics were extracted from online databases (https://shigen.nig.ac.jp/rice/oryzabase/ and https://rapdb.dna.naro.go.jp/) (**Supplementary Table 3**). Among the genes listed, only two (*OsRbs5* and *OsLAC11*) are directly mentioned as related to kernel smut resistance in the trait ontology term. The *OsRbs5* gene is involved in the assembly and stability of the 40S ribosomal subunit and plays a vital role in lipid metabolism, while the *OsLAC11* gene is essential for lignin degradation and the detoxification of lignin-derived compounds (**Supplementary Table 3**). An organ-specific gene expression analysis revealed that the *OsLAC11* gene was highly expressed during the first flowering day (**Figure 2**).

**Figure 3:**
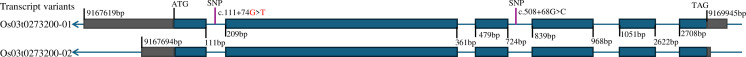
Transcript variants of the *OsLAC11* gene. The single-nucleotide polymorphism (SNP) position is mentioned based on Ogino et al. (Ogino et al., 2007).

**Figure 4:**
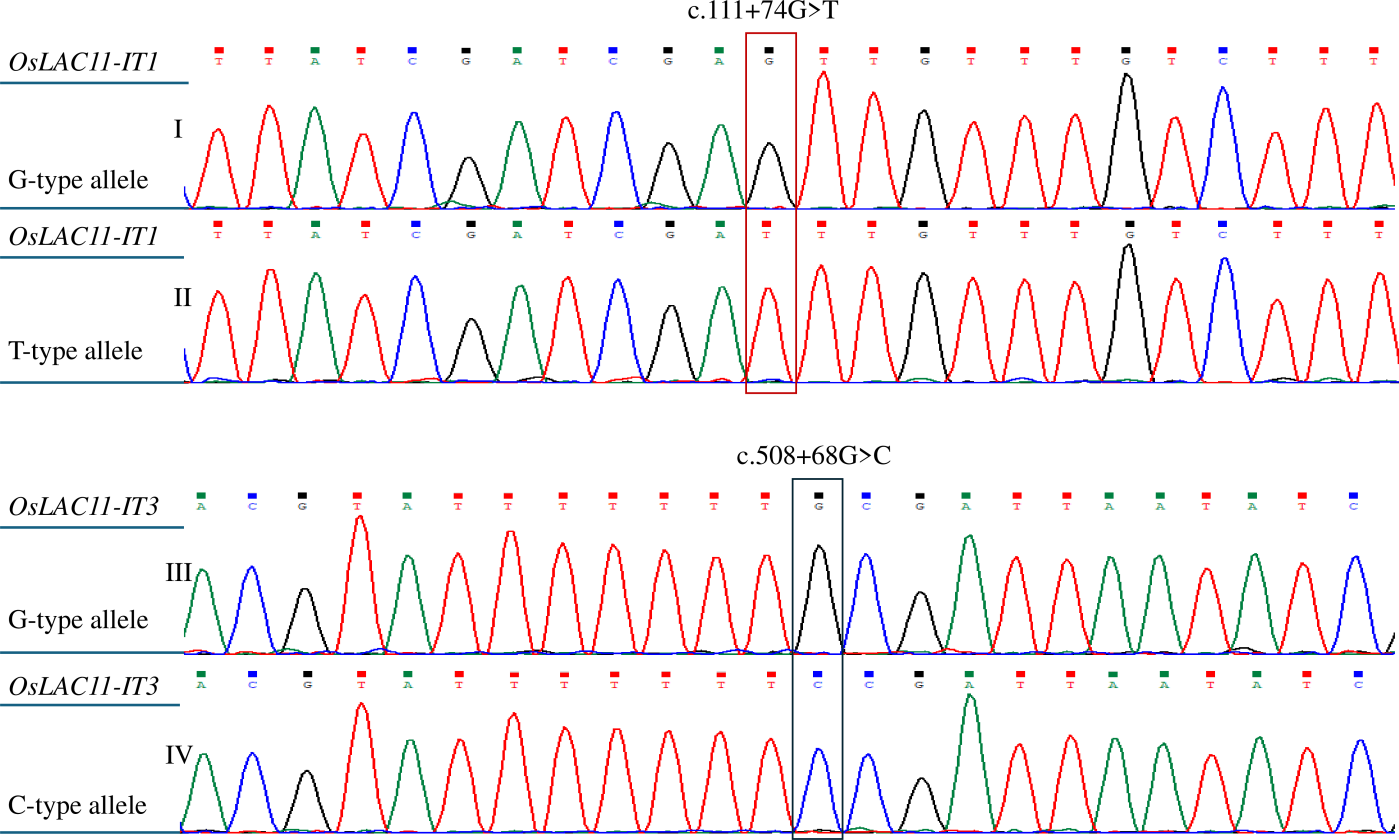
Allelic variations of the *OsLAC11* gene in different rice genotypes. **(I)** chromatogram of G-type allele; **(II)** chromatogram of T-type allele; **(III)** chromatogram of G-type allele; and **(IV)** chromatogram of C-type allele. IT is part of the intron of the *OsLAC11* gene.

The *OsLAC11* gene (*Os03g0273200*), also referred to as *LAC11* or *Lac11*, encodes a laccase enzyme (EC 1.10.3.2), known as LACCASE 11. The *OsLAC11* gene has two types of transcript variants (**Figure 3**). Sequence alignment analysis revealed the presence of two SNPs in the *OsLAC11* gene: one located in the first intron (c.111 + 74G>T) and another in the third intron (c.508 + 68G>C) (**Figures 3**, **4**). All bases in the *OsLAC11* gene across the selected rice lines were similar, except for SNPs (**Figure 4**), making it suitable for SNP-based marker development.

### 3.2 Functional marker analysis and validation

**Figure 5:**
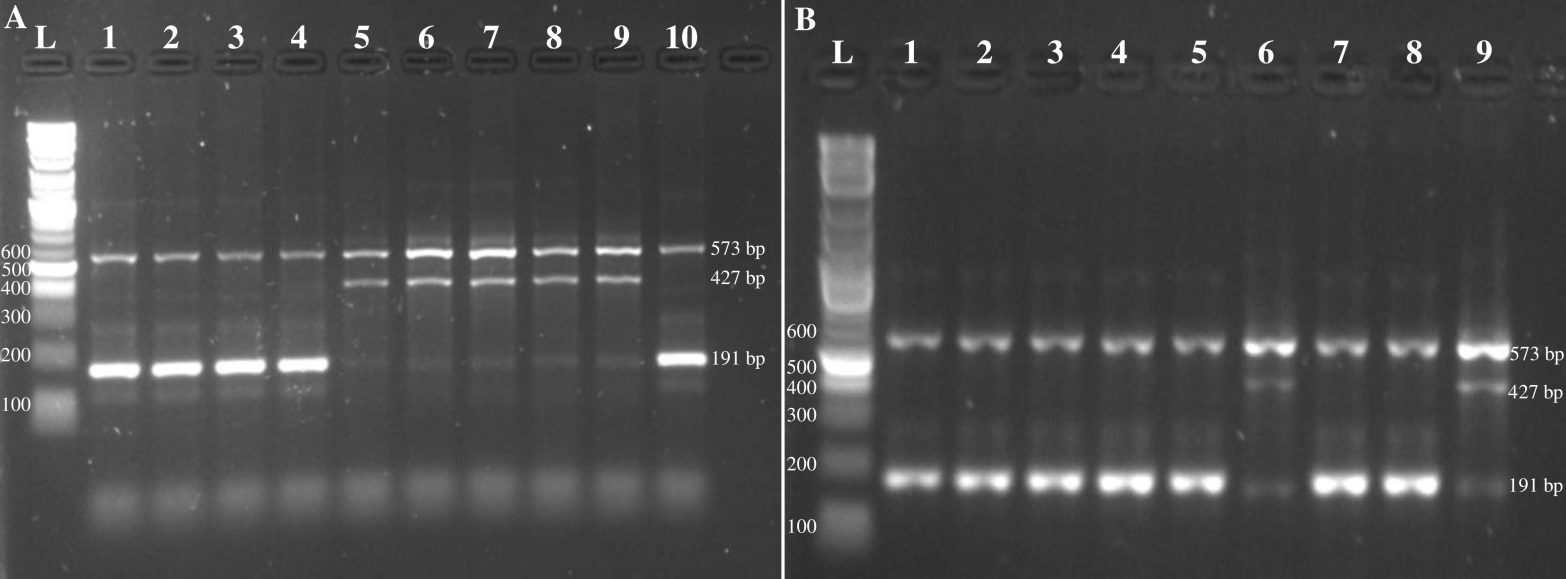
Genotypic analysis of different rice cultivars using the kernel smut marker (KS Marker) and validation using different rice lines. A; L, 1kb^+^ Ladder; Lane 1, Saturn; Lane 2, Arkrose; Lane 3, Vista; Lane 4, Zenith; Lane 5, Kangni; Lane 6, Dokri Basmati; Lane 7, Basmati 370; Lane 8, Sada Gulab; Lane 9, Basmati; Lane 10, Nipponbare (genomic reference). B; L, 1kb^+^ Ladder; Lane 1, Saturn; Lane 2, Roy J; Lane 3, Addi Jo; Lane 4, Thad; Lane 5, Titan; Lane 6, Kangni Basmati; Lane 7, Trinity; Lane 8, Presidio; Lane 9, Basmati-370. Band 573 bp is common for all, indicating successful amplification; Band 427 bp is specific to susceptible genotypes; Band 191 bp is specific to resistant genotypes.

Genotypic analysis of functional markers for the *OsLAC11* gene in nine selected cultivars and Nipponbare (genomic reference) revealed three distinct band types (**Figure 5A**). The presence of a 573 bp band, which includes the region containing the G/T SNP, was common to all genotypes, indicating successful amplification. The 191 bp band, which shows the G-type SNP, was present only in the kernel smut-resistant genotypes, while the 427 bp band for the T-SNP was present only in the susceptible genotypes. During marker validation across six rice cultivars tested with artificial kernel smut inoculation in the field over two years, similar banding patterns were observed (**Figure 5B**).

### 3.3 Phenotypic evaluation for kernel smut resistance

The phenotypic assessment and infection scores for kernel smut disease are presented in **Table 1**. Four rice cultivars (Saturn, Arkrose, Vista, and Zenith) had very low infection rates (<0.8%), whereas Dokri Basmati exhibited a substantially higher infection rate (<7.3%). Six rice cultivars were evaluated for two years (2023 and 2024) in the field (**Table 1**), and responses to kernel smut infection were consistent across both years: resistant (Addi Jo and Roy J), intermediate (Thad and Titan), and susceptible (Presidio and Trinity). Among the 16 cultivars investigated in this study, 14 showed phenotypic results consistent with the marker analyses. However, two rice cultivars (Presidio and Trinity) were phenotypically susceptible despite showing a 191 bp band in genotyping that is associated with resistant genotypes.

**Table 1:** Genotypic and phenotypic kernel smut ratings of rice cultivars evaluated from a field disease nursery in Beaumont, Texas, in 2023 and 2024.

Cultivar	Infection rate (%) in 2023	Infection rate (%) in 2024	Phenotypic rating	Genotypic rating
Saturn		< 0.4	Resistant	GG
Arkrose		< 0.3	Resistant	GG
Vista		< 1	Resistant	GG
Zenith		< 0.8	Resistant	GG
Kangni Basmati		< 2.5	Intermediate	TT
Dokri Basmati		< 7.3	Susceptible	TT
Basmati 370		0.00	Not detected †	TT
Sada Gulab		0.00	Not detected †	TT
Basmati		0.00	Not detected †	TT
Addi Jo	< 1	< 2	Resistant/Intermediate	GG
Roy J	< 1	< 1	Resistant	GG
Thad	< 3	< 3	Intermediate	TT
Titan	< 3	< 2	Intermediate	TT
Presidio	> 5	> 5	Susceptible	GG
Trinity	> 5	> 5	Susceptible	GG

†Due to the extended flowering period, plants were inoculated too late and showed no response. Infection score > 3% is classified as susceptible, < 3% as intermediate (with 3% being the maximum smutted grain allowed by the USDA standards), and < 1% as resistant. GG for Resistance; TT for Susceptible or Intermediate.

## 4 Discussion

Rice kernel smut is one of the most significant grain diseases, threatening rice production globally. This disease predominantly impacts grain quality rather than yield. Although the severity and incidence of kernel smut are rising, the identification of resistant varieties and the investigation of management strategies remain limited. The use of resistant cultivars is the most effective strategy for managing kernel smut. Identifying resistance genes and developing reliable molecular markers are critical first steps toward achieving this goal. This study identified a candidate gene associated with kernel smut using gene expression databases, transcriptomic data, and scientific literature, and reports the development of the first functional molecular marker for this resistance. The marker’s utility was demonstrated through its application in rice genotyping and phenotypic validation.

### 4.1 *Tilletia horrida* effector highlights the responsible gene for kernel smut

Plant pathogenic fungi secrete numerous proteins, but only a few have been identified as effectors (Wang et al., 2019; Kainat et al., 2025). Conversely, plant receptor proteins that activate defense responses can detect these pathogen effectors (Wang et al., 2019; Kainat et al., 2025). Therefore, identifying effectors beyond the host is essential for non-host resistance during attempts of inoculation by non-host pathogens (Stam et al., 2014; Kainat et al., 2025). For example, ThSCSP_12 in *Tilletia horrida* induces cell death and defense response in non-host plant *N. benthamiana* (Zhang et al., 2020; Shu et al., 2022). A secreted cell-surface protein, ThCSP5, functions as an elicitor that activates plant immunity and enhances resistance against *Tilletia horrida* (Shu et al., 2025a). Research indicates that secretory proteins from plant pathogens function as effectors or elicitors during pathogen-host interactions. These effectors are frequently detected within plant defense signaling pathways (Wang et al., 2019; Kainat et al., 2025). The *OsnTNB.11* gene, encoding an NB-LRR protein, positively regulates rice kernel smut resistance by modulating the ethylene signaling pathway (Shu et al., 2025b). Based on genome sequencing, *T. horrida* encodes 597 secreted proteins, of which 131 are predicted as effectors (Wang et al., 2018a, 2019). Two potential effectors (smut_5844 and smut_2965) were identified by transient expression in *Nicotiana benthamiana* (Georg.) as producing necrotic phenotypes, and their encoded genes were up-regulated during early infection. The encoded proteins were also proven to be secreted using a yeast secretion system. To induce non-host cell death, the putative effector gene smut_5844 requires a signal peptide, whereas smut_2965 requires ribonuclease catalytic active sites. Thus, in this study, the effector smut_5844 was targeted as it induces necrosis in *Nicotiana benthamiana*. The gene responsible for smut_5844 effector was identified as the laccase-10 protein (*OsLAC10*), predicted to be involved in plant lignification and iron metabolism (Wang et al., 2019; Shu et al., 2022). In the current study, the protein sequence of smut_5844, referred to as *LOC_Os03g16610* (*OsLAC10*), was analyzed for homology using the Basic Local Alignment Search Tool (BLAST) against multiple databases. These analyses showed high similarity to the rice gene *Os03g0273200* (*LAC11*, *Lac11*, or *OsLAC11*), which encodes the protein laccase (EC 1.10.3.2; LACCASE 11 or laccase 11). The *OsLAC11* gene has two transcript variants that produce distinct mRNA products and support different pre-mRNA maturation (splicing) pathways via alternative splicing sites. Among the genes reported, only the *OsRbs5* and *OsLAC11* genes are directly associated with kernel smut resistance within the trait ontology term. In addition, the organ-specific gene expression analysis indicated that the *OsLAC11* gene was highly expressed during the first day of flowering. Overall, these findings indicate that the *OsLAC11* gene is highly effective for kernel smut resistance and could be used to develop a kernel smut marker.

### 4.2 Gene sequencing for SNP marker development

Targeted gene sequence analysis is a powerful technique used in rice research to identify genetic variations in specific genes associated with important traits (Sun et al., 2022). In the current investigation, DNA sequence alignment analysis of the *OsLAC11* gene in nine rice germplasms that were selected from previously published literature (Akhtar and Sarwar, 1988; Singh et al., 1998) revealed two allelic variants: one in the first intron (c.111 + 74G>T) and another in the third intron (c.508 + 68G>C). Although both SNPs occur within intronic regions, they may still influence gene regulation or post-translational modification.

The SNP present in the first intron was selected for marker development because the first intron has been recognized for its significant role in both transcriptional and translational regulation. The first intronic DNA sequence features a unique epigenetic marker and nucleosome density, which determine the transcription start site and the elongation of its expression (intron-mediated enhancement), translation control in the 5´-UTR, and trans-acting function after transcription (Zalabák and Ikeda, 2020; Salih et al., 2021; Gong et al., 2023). Previous studies indicated that the base substitution (G → T) at loci of the first intron (In1) of the *Waxy* (*Wx*) gene results in decreased amylose content, and the SNP selection of this locus helps to breed cultivars with desired amylose content (Cai et al., 2015; Pariasca-Tanaka et al., 2025). The SNP typing method was based on the PCR-CTPP, and its practicality was confirmed through testing 23 cultivars with known SNPs and amylose content (Cai et al., 2015). Building on this well−established framework, we designed and developed a functional marker targeting the first intron SNP of the *OsLAC11* for kernel smut resistance.

### 4.3 *T. horrida* infection and proposed kernel smut resistance mechanisms

**Figure 6:**
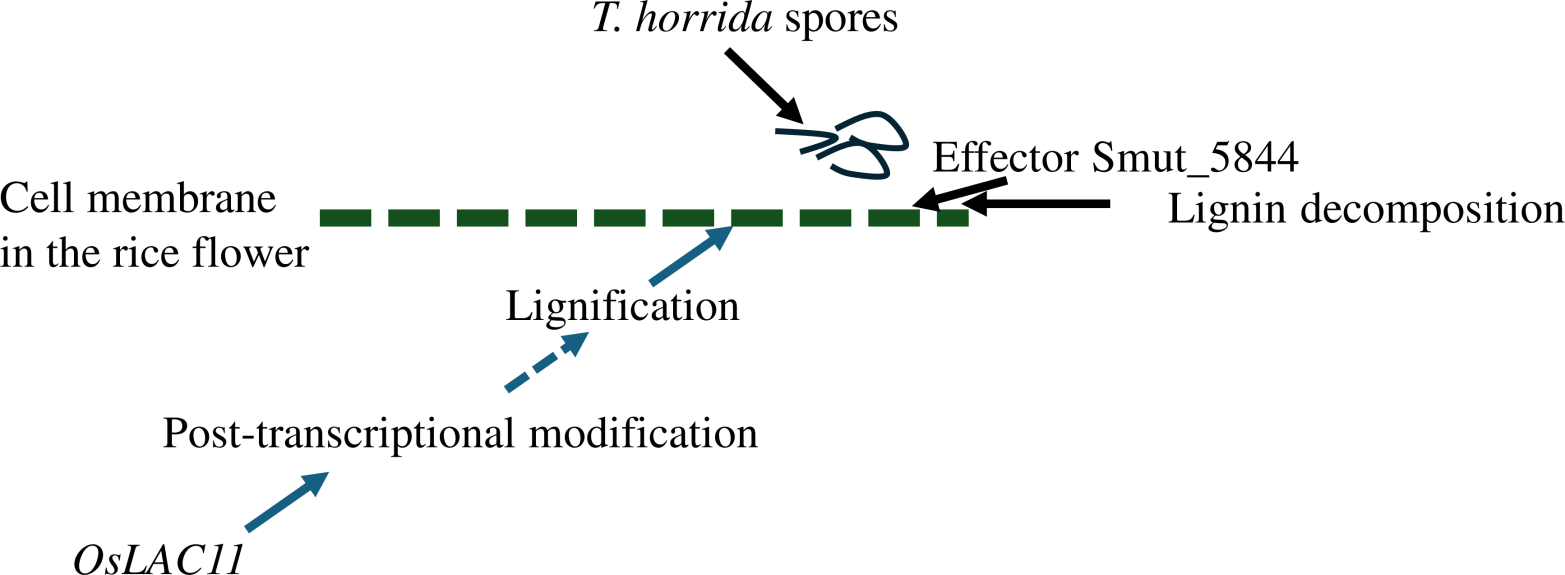
Hypothetical mechanism for kernel smut resistance in rice. The *T. horrida* spore infects and stays on the surface of the cell membrane of the rice flower. During the infection, *T. horrida* releases *Smut_5844* effector, which can initiate lignin decomposition in susceptible rice lines. On the other hand, the *OsLAC11* protein undergoes post-translational modification to enhance lignification in the resistant rice lines.

During infection, rice cells trigger PAMP-triggered immunity (PTI), a nonspecific immune response. When the fungus releases PAMPs and effectors to overcome PTI in rice, specific effectors are recognized by homologous NLR proteins. In resistant rice plants, this recognition activates a strong immune response called effector-triggered immunity (ETI) (Cui et al., 2015). Both PTI and ETI trigger similar immune responses, such as calcium influx, transcriptional reprogramming, and hormone regulation (Cui et al., 2015; Wang et al., 2020c). PTI and ETI are closely linked and mutually reinforce one another, thereby conferring strong disease resistance (Yuan et al., 2021). Based on an effector protein for kernel smut (*smut_5844*) (Wang et al., 2019) and subsequent analysis, this study highlights *OsLAC11* as a potential candidate gene for kernel smut resistance. The *OsLAC11* plays a critical role in lignification, helps maintain cell wall structure and mechanical strength, supports plant responses to environmental stressors and defense mechanisms, promotes wound healing, facilitates iron metabolism, and contributes to the polymerization of phenolic compounds. The ability of plant laccases to oxidize lignin precursors highlights their role in lignification of the plant cell wall. These laccase enzymes are released into the apoplast, where they contribute to lignin biosynthesis and the repair of damaged plant tissues (Berthet et al., 2012; Janusz et al., 2020). In contrast, fungal laccases primarily facilitate the degradation of lignocellulose polymers and contribute to fungal defense, virulence, pathogenesis, pigmentation, and sporulation. Their primary function is to facilitate the biodegradation of lignocellulose, thereby supporting the carbon cycle in the biosphere (Liu et al., 2017; Gupta et al., 2025). These contrasting functions facilitate speculation of a hypothetical mechanism that describes *T. horrida* infection and the rice plant response mediated by the *OsLAC11* gene (**Figure 6**). Beyond the effector-host protein interactions, transcription factors (TFs) also play significant roles in regulating plant defense responses and secondary metabolism. For instance, recent genome-wide characterization of TF families has identified the *WOX* gene family in the ramie plant, which is involved in regulating plant development and stress responses (Abubakar et al., 2023). Similarly, another study revealed that R2R3-MYB TFs, such as PgMYB2 in *Panax ginseng*, positively regulate dammarenediol synthase expression, thereby influencing triterpene saponin biosynthesis (Liu et al., 2019). This suggests that the *OsLAC11* gene identified in our study may be regulated by upstream transcription factors in response to *T. horrida* infection, indicating the need for further investigation into the regulatory network governing lignification-mediated resistance.

### 4.4 Phenotypic evaluation for the marker validation

To date, the only method for identifying resistant and susceptible rice genotypes has been artificial inoculation at the booting stage (3 to 5 days before heading), followed by quantification of *T. horrida* infection in rice kernels (Wang et al., 2018c; Khanal, 2024). Several rice cultivars have been evaluated and scored for their reactions to kernel smut disease (**Supplementary Table 4**).

In this study, nine rice cultivars were evaluated for kernel smut resistance and subsequently selected for sequencing to identify allelic variants, with each having been tested for kernel smut resistance by previous researchers (Templeton and Johnston, 1970; Akhtar and Sarwar, 1988). A 10^th^ cultivar, Nipponbare, was selected as the genome reference (reference rice variety) to detect the SNP and to validate the genotyping accuracy and sequencing result (Chen et al., 2014; Shang et al., 2023). Depending on the presence of SNPs, a molecular marker has been designed and used to assess the genotypic status of the six selected rice cultivars. These six cultivars were selected from artificial kernel smut inoculation experiments conducted in 2023 and 2024. Nearly all tested cultivars showed similar results in both phenotypic and marker analyses, indicating that phenotypic data support and confirm the genotypic findings. However, two exceptions were observed: Presidio and Trinity displayed susceptibility in the phenotypic evaluation but exhibited a 191 bp band associated with resistant genotypes. This may be due to differential post-transcriptional modification and/or to other genes influencing *OsLAC11* expression (Abubakar et al., 2023). Another contributing factor may be the uneven nitrogen application during hand broadcasting, as elevated levels of nitrogen are known to increase susceptibility to kernel smut under favorable environmental conditions (Kalboush et al., 2018).

## 5 Conclusion

Based on publicly available gene expression datasets, transcriptomic data, and hypothesized genes related to kernel smut disease in databases and published literature, the current research identified the *OsLAC11* gene as the most likely candidate gene responsible for kernel smut resistance in rice and proposed that the marker can be utilized in marker-assisted selection. Sanger sequencing and alignment analysis of the *OsLAC11* gene revealed two single-nucleotide polymorphisms (SNPs) within the intronic region. One potential SNP (G/T allele) located in the first intron was selected for the development of functional markers using polymerase chain reaction with two pairs of confronting primers. This putative functional marker effectively distinguished resistant from susceptible rice cultivars, as validated by artificial inoculation and phenotypic scoring. This study is the first report of a putative marker for kernel smut resistance and its application in rice genotyping, with accompanying phenotypic validation. Future work will include functional and proteomic analyses of the *OsLAC11* gene to confirm its role in resistance to kernel smut in rice. This study recommended Saturn, Arkrose, Vista, Zenith, Addi Jo, and Roy J cultivars as donor parents for kernel smut resistance. These cultivars are also being incorporated into the development of kernel smut-resistant rice cultivars.

## Data Availability

Publicly available datasets were analyzed in this study. This data can be found here: https://shigen.nig.ac.jp/rice/oryzabase/ and https://rapdb.dna.naro.go.jp/.
