# Supplementary material for: A functional PCR-CTPP marker targeting an intronic SNP in OsLAC11 gene for detecting kernel smut resistance in rice
Source: Front Plant Sci. 2026 Jul 7;17:1809531. doi: 10.3389/fpls.2026.1809531 (PMC13385412; doi:10.3389/fpls.2026.1809531)
Supplement: Supplementary file 1 [file Table1.docx]

Supplementary Material

# Supplementary Tables

**Supplementary Table 1** List of rice cultivars used in this study.

| No. | **Rice cultivar** | **Origin** | **Accession number** | **Gene Bank or Source** |
| --- | --- | --- | --- | --- |
| 1 | Saturn | Louisiana, USA | CIor 9540 | NSGC |
| 2 | Arkrose | Arkansas, USA | CIor 8310 | NSGC |
| 3 | Vista | Louisiana, USA | CIor 12346 | NSGC |
| 4 | Zenith | Arkansas, USA | PI 389996 | NSGC |
| 5 | Kangni | Sindh, Pakistan | PI 392158 | NSGC |
| 6 | Dokri Basmati | Sindh, Pakistan | PI 431147 | NSGC |
| 7 | Basmati 370 | Pakistan | PI 439018 | NSGC |
| 8 | Sada Gulab | Sindh, Pakistan | PI 392114 | NSGC |
| 9 | Basmati | Sindh, Pakistan | PI 412839 | NSGC |
| 10 | Nipponbare | Aiti, Japan | PI 514663 | NSGC |
| 11 | Trinity | Texas A&M AgriLife Research Center, Beaumont, TX, USA | PI 700309 | Texas A&M University, TX, USA |
| 12 | Presidio | Texas, USA | PI 636465 | NSGC |
| 13 | Addi Jo | Louisiana State University Agricultural Center, Crowley, LA, USA | PI 701526 | Louisiana State University, LA, USA |
| 14 | Roy J | University of Arkansas System, Fayetteville, AR, USA | PI 660665 | Arkansas Crop Variety Improvement Program, AR, USA |
| 15 | Thad | Mississippi State University, MS, USA | RU9804054 | Mississippi Agricultural and Forestry Experiment Station, MS, USA |
| 16 | Titan | University of Arkansas System, Fayetteville, AR, USA | PI 680613 | Arkansas Crop Variety Improvement Program, AR, USA |

Abbreviations: NSGC: National Small Grains Collection, USDA-ARS, Aberdeen, Idaho, USA

**Supplementary Table 2** List of Sanger sequencing primers and kernel smut marker for the *OsLAC11* gene.

| Assay Set | **Type** | **Sequence** | **Start** | **Length** | **Tm**  **(50mM; ℃)** | **GC (%)** | **Amplicon (bp)** |
| --- | --- | --- | --- | --- | --- | --- | --- |
| Assay Set 1 | Forward Primer | CTCGCATTGCTTCTCCTCTATG | 16 | 22 | 62.00 | 50 | 573 |
|  | Reverse Primer | CCCGTTGATGGTGAAGTTGTA | 568 | 21 | 62.00 | 48 |  |
| Assay Set 2 | Forward Primer | CAGAGCTACGTGTACAACTTCA | 555 | 22 | 62.00 | 45 | 543 |
|  | Reverse Primer | CCGGAGCAGGTACATCTTTC | 1078 | 20 | 62.00 | 55 |  |
| Assay Set 3 | Forward Primer | GCAGCCCGGAAAGATGTA | 1070 | 18 | 62.00 | 55 | 695 |
|  | Reverse Primer | TCGACACGTTGGTGTTGTT | 1746 | 19 | 62.00 | 47 |  |
| Assay Set 4 | Forward Primer | CTGGAGCCGTTCAACTACAC | 1713 | 20 | 62.00 | 55 | 527 |
|  | Reverse Primer | CTAGCACATGGGAAGATCGG | 2220 | 20 | 62.00 | 55 |  |
| KERNEL SMUT_R_In1 | KS_R_In1_1F | CTCGCATTGCTTCTCCTCTATG | 16 | 22 | 62.00 | 50 | 191 |
|  | KS_R_In1_1R_1 | AAACAGCCAAAGACAAACAAC | 186 | 22 | 59.70 | 41 |  |
| KERNEL SMUT_S_In1 | KS_S_In1_2F_2 | CGTTTGGTTATCTTATCGATCGAT | 162 | 24 | 60.50 | 38 | 427 |
|  | KS_S_In1_2R | CCCGTTGATGGTGAAGTTGTA | 568 | 21 | 60.80 | 48 |  |

**Supplementary Table 3** Genetic characteristics of selected genes for kernel smut resistance.

| **Gene symbol** | **Gene ID** | **Gene Ontology (GO)** | **Trait Ontology (TO)** | **Gene Function** | **Reference** |
| --- | --- | --- | --- | --- | --- |
| *OsRbs5* | *Os07g0616600* | GO:0050832 - defense response to fungus | TO:0000048 - kernel smut disease resistance | Required for the assembly and stability of the 40S ribosomal subunit.  Plays a crucial role in lipid metabolism. | Wang et al. (2015)  Wang et al (2018a)  Wang et al (2019)  Liu et al (2016) |
| *OsLAC10* | *Os02g0749700* | GO:0046688 - response to copper ion | TO:0000021 - copper sensitivity | Response to environmental stressors, particularly in copper toxicity | Liu et al. (2017) |
| *OsLAC11* | *Os03g0273200* | GO:0050832 - defense response to fungus | TO:0000048 - kernel smut disease resistance | Lignin degradation and detoxification of lignin-derived products | Wang et al. (2015) |
| *OsWRKY70* | *Os05g0474800* | GO:0050832 - defense response to fungus | TO:0002657 - oxidative stress | Enhancing stress tolerance | Kantama et al. (2013) |
| *OsWRKY12* | *Os01g0624700* | GO:0006952 - defense response | TO:0000112 - disease resistance | Defense responses against biotic and abiotic stresses | Kantama et al. (2013)  Choi et al. (2017)  Wang et al. (2020) |
| *OsWRKY24* | *Os01g0826400* | GO:0006952 - defense response | TO:0000172 - jasmonic acid sensitivity | Regulating grain size, tolerance, and resistance to stress | Li et al. (2023) |
| *OsCML7* | *Os08g0117400* | GO:0005509 - calcium ion binding |  | Transduce the increase in cytosolic Ca^2+^ concentrations | Chinpongpanich et al. (2012) |
| *OsCML14* | *Os05g0577500* | GO:0050832 - defense response to fungus | TO:0000468 - leaf blast disease resistance | Transduce the increase in cytosolic Ca^2+^ concentrations | Gottin et al. (2021)  Liu et al. (2023) |
| *Osrboh9* | *Os12g0541300* | GO:0050832 - defense response to fungus | TO:0000439 - fungal disease resistance | Regulate rice seed vigor and response to environmental stressors | Chen et al. (2024) |
| *OsRR1* | *Os04g0442300* | GO:2000028 - regulation of photoperiodism, flowering | TO:0000137 - days to heading | Response to environmental stressors and overall growth and development | Hu et al. (2023) |
| *OsABF1* | *Os01g0867300* | GO:0010187 - negative regulation of seed germination | TO:0000430 - germination rate | Response to abiotic stress, hormonal signaling, and delayed flowering | Tang et al. (2021) |
| *OsCTR2* | *Os02g0527600* | GO:0048573 - photoperiodism, flowering | TO:0002616 - flowering time | Regulate tiller formation and flowering time | Wang et al. (2013) |

**Supplementary Table 4** List of rice cultivars previously tested for the kernel smut disease reaction.

| **Rice cultivar** | **Reaction** | **Country** | **References** |
| --- | --- | --- | --- |
| Sathra-278, Ratria,  Mushakan, Melhar-364, Jhona-349,  Motia, Bara,  Red Rice, Kangni,  Palman, Basmati Pak, and  Dokri Basmati | S | Pakistan | Akhtar and Sarwar (1988) |
| Sada Gulab and Basmati-370 | I |  |  |
| IR-579 and C-622 | R |  |  |
| 4766A | HR | Hubei, China | Wang et al. (2018b) |
| JuFeng2A | R |  |  |
| JiangCheng3A | R |  |  |
| TianFengA | MR |  |  |
| Elon-elon, Mancasar str. 3 | R | Philippines | Singh et al. (1998) |
| Vista, Arkrose, Zenith, Saturn, and Cl 9647 | R | USA | Templeton and Johnston (1970) |

*S = Susceptible; R = Resistant; HR = Highly Resistant; MR = Moderately Resistant; and I = Intermediate.

**References**

Akhtar, M., and Sarwar, M. (1988). Rice cultivars resistance to kernel smut. *Pakistan Journal of Agricultural Research* 9(2)**,** 266-267.

Chen, Y., Zhang, R., Wang, R., Li, J., Wu, B., Zhang, H., et al. (2024). Overexpression of *OsRbohH* enhances heat and drought tolerance through ROS homeostasis and ABA mediated pathways in rice (*Oryza sativa* L.). *Plants* 13(17)**,** 2494. doi: https://doi.org/10.3390/plants13172494.

Chinpongpanich, A., Limruengroj, K., Phean-O-Pas, S., Limpaseni, T., and Buaboocha, T. (2012). Expression analysis of calmodulin and calmodulin-like genes from rice, *Oryza sativa* L. *BMC Research Notes* 5(1)**,** 625. doi: https://doi.org/10.1186/1756-0500-5-625.

Choi, N.Y., Lee, E., Lee, S.G., Choi, C.H., Park, S.R., Ahn, I., et al. (2017). Genome-wide expression profiling of *OsWRKY* superfamily genes during infection with *Xanthomonas oryzae* pv. oryzae using real-time PCR. *Frontiers in Plant Science* 8**,** 1628. doi: https://doi.org/10.3389/fpls.2017.01628.

Gottin, C., Dievart, A., Summo, M., Droc, G., Périn, C., Ranwez, V., et al. (2021). A new comprehensive annotation of leucine‐rich repeat‐containing receptors in rice. *The Plant Journal* 108(2)**,** 492-508. doi: https://doi.org/10.1111/tpj.15456.

Hu, X., Yu, P., Zhang, Y., Gao, Z., Sun, B., Wu, W., et al. (2023). Mutation of *DEFECTIVE EMBRYO SAC1* results in a low seed-setting rate in rice by regulating embryo sac development. *Journal of Experimental Botany* 74(5)**,** 1501-1516. doi: https://doi.org/10.1093/jxb/erac506.

Kantama, L., Junbuathong, S., Sakulkoo, J., de Jong, H., and Apisitwanich, S. (2013). Epigenetic changes and transposon reactivation in Thai rice hybrids. *Molecular Breeding* 31(4)**,** 815-827. doi: https://doi.org/10.1007/s11032-013-9836-x.

Li, J., Chen, Y., Zhang, R., Wu, B., and Xiao, G. (2023). Expression identification of three *OsWRKY* genes in response to abiotic stress and hormone treatments in rice. *Plant Signaling & Behavior* 18(1)**,** 2292844. doi: https://doi.org/10.1080/15592324.2023.2292844.

Liu, J., Zhang, C., Wei, C., Liu, X., Wang, M., Yu, F., et al. (2016). The RING finger ubiquitin E3 ligase *OsHTAS* enhances heat tolerance by promoting H_2_O_2_-induced stomatal closure in rice. *Plant Physiology* 170(1)**,** 429-443. doi: https://doi.org/10.1104/pp.15.00879.

Liu, Q., Luo, L., Wang, X., Shen, Z., and Zheng, L. (2017). Comprehensive analysis of rice laccase gene (*OsLAC*) family and ectopic expression of *OsLAC10* enhances tolerance to copper stress in *Arabidopsis*. *International Journal of Molecular Sciences* 18(2)**,** 209. doi: https://doi.org/10.3390/ijms18020209.

Liu, W.Z., Li, Z.Y., Liu, C., Yu, X.T., Yu, W.Q., and Li, P. (2023). *Paenibacillus terrae* NK3-4 regulates the transcription of growth-related and stress resistance-related genes in rice. *Genome* 66(6)**,** 131-149. doi: https://doi.org/10.1139/gen-2022-0072.

Singh, R., Dodan, D., and Sheoran, O. (1998). Kernel Smut of rice: present status. *International Journal of Tropical Plant Diseases* 16(2)**,** 149-168.

Tang, L., Xu, H., Wang, Y., Wang, H., Li, Z., Liu, X., et al. (2021). *OsABF1* represses gibberellin biosynthesis to regulate plant height and seed germination in rice (*Oryza sativa* L.). *International Journal of Molecular Sciences* 22(22)**,** 12220. doi: https://doi.org/10.3390/ijms222212220.

Templeton, G., and Johnston, T. (1970). A source of resistance to rice kernel smut organism. *Rice* 73**,** 74.

Wang, A., Pan, L., Niu, X., Shu, X., Yi, X., Yamamoto, N., et al. (2019). Comparative secretome analysis of different smut fungi and identification of plant cell death-inducing secreted proteins from *Tilletia horrida*. *BMC Plant Biology* 19**,** 1-14. doi: https://doi.org/10.1186/s12870-019-1924-6.

Wang, A., Shu, X., Niu, X., Zhao, W., Ai, P., Li, P., et al. (2018a). Comparison of gene co-networks analysis provide a systems view of rice (*Oryza sativa* L.) response to *Tilletia horrida* infection. *PLoS One* 13(10)**,** e0202309. doi: https://doi.org/10.1371/journal.pone.0202309.

Wang, A., Yin, D., Fu, R., Pan, L., Gu, S., Jiang, B., et al. (2018b). Evaluation of resistance to rice kernel smut in seventy-eight species of rice sterile Line. *Acta Phytopathol* 48(3)**,** 297-304.

Wang, A., Zha, Z., Yin, D., Shu, X., Ma, L., Wang, L., et al. (2020). Comparative transcriptome analysis of *Tilletia horrida* infection in resistant and susceptible rice (*Oryza sativa* L.) male sterile lines reveals potential candidate genes and resistance mechanisms. *Genomics* 112(6)**,** 5214-5226. doi: https://doi.org/10.1016/j.ygeno.2020.09.036.

Wang, H., Niu, Q.W., Wu, H.W., Liu, J., Ye, J., Yu, N., et al. (2015). Analysis of non‐coding transcriptome in rice and maize uncovers roles of conserved lnc RNA s associated with agriculture traits. *The Plant Journal* 84(2)**,** 404-416. doi: https://doi.org/10.1111/tpj.13018.

Wang, Q., Zhang, W., Yin, Z., and Wen, C.-K. (2013). Rice *CONSTITUTIVE TRIPLE-RESPONSE2* is involved in the ethylene-receptor signalling and regulation of various aspects of rice growth and development. *Journal of Experimental Botany* 64(16)**,** 4863-4875. doi: https://doi.org/10.1093/jxb/ert272.
